# Impact of a silver layer on the membrane of tap water filters on the microbiological quality of filtered water

**DOI:** 10.1186/1471-2334-8-133

**Published:** 2008-10-08

**Authors:** Ralf-Peter Vonberg, Dorit Sohr, Juliane Bruderek, Petra Gastmeier

**Affiliations:** 1 Institute for Medical Microbiology and Hospital Epidemiology, Medical School Hannover, Germany; 2 Institute for Hygiene and Environmental Medicine, Charité – University Medicine Berlin, Germany

## Abstract

**Background:**

Bacteria in the hospital's drinking water system represent a risk for the acquisition of a nosocomial infection in the severely immunocompromised host. Terminal tap water filters may be used to prevent nosocomial Legionnaires' disease. We present data from water samples using an improved kind of tap water filters.

**Methods:**

In a blinded study on an intermediate care unit of the thoracic surgery department, a modified type of the Germlyser water filter (Aqua-Free Membrane Technology) with a newly-introduced silver layer on the filtration membrane was compared to its preceding type without such a layer on 15 water outlets. We determined growth of *Legionella*, other pathogenic bacteria, and the total heterotrophic plate count in unfiltered water and filtered water samples after filter usage intervals of 1 through 4 weeks.

**Results:**

A total of 299 water samples were tested. Twenty-nine of the 60 unfiltered water samples contained *Legionella *of various serogroups (baseline value). In contrast, all samples filtered by the original water filter and all but one of the water samples filtered by the modified filter type remained *Legionella*-free. No other pathogenic bacteria were detected in any filtered sample. The total plate count in water samples increased during use of both kinds of filters over time. However, for the first 7 days of use, there were significantly fewer water samples containing >100 CFU per mL when using the new filter device compared with the older filters or taps with no filter. No advantage was seen thereafter.

**Conclusion:**

The use of this type of terminal water filter is an appropriate method to protect immunocompromised patients from water-borne pathogens such as *Legionella*.

## Background

*Legionella *spp. are commonly detected in hospital water supply systems [1,2]. Especially in the severely immunocompromised host, such as patients after bone marrow or solid organ transplantation, these pathogens are well-know causative agents of life-threatening nosocomial infections [3-5]. The minimal number of *Legionella *needed to actually cause disease remains unknown. Infection in susceptible individuals may occur at concentrations <1 colony forming unit (CFU) per mL [6]. That is why the use of pathogen-free water for high risk patient care is recommended by the Healthcare Infection Control Practices Advisory Committee (HICPAC) of the Centers for Disease Control and Prevention (CDC), by the Infectious Diseases Society of America (IDSA), and by the American Society of Blood and Marrow Transplantation (ASBMT) [7,8].

Hannover Medical School is a tertiary university hospital and each year several hundreds of patients at high risk for water-borne nosocomial infections are cared for in our facility. Eradication of *Legionella *from our water system is a problematic issue. Copper-silver ionization [9] and hyperchlorination [10] may be possible methods for the reduction of *Legionella*, but both of these approaches are hardly options in our facility due to the German drinking water regulation law [11]. Superheat and consecutive flushing of the complete plumbing systems at temperatures >73°C [12] demands enormous logistic effort to reduce the risk of scalding on the user's side. Further disadvantages of superheat-and-flush are the cost for energy and possible damage to the plumbing system due to high temperatures. As for ozone treatment [13] and UV radiation [14], long-term success has rarely been reported with superheat-and-flush.

If eradication of *Legionella *cannot be accomplished, exposition prophylaxis (avoiding contact) may serve as an alternative. Purchase of sterile water [15] for high risk patient care is a quite expensive option and becomes inconvenient for treatment of longer time periods. Therefore we chose the option of point-of-use water filtration [16,17]. Water filters, too, are an expensive infection control measure, but prolonged usage intervals before the filters are changed may reduce the costs noticeably. Retrograde bacterial contamination is a well-known problem in the use of tap water filters and limits their usage interval [18]. To extend the usage time of such filters one manufacturer has developed a silver layer on the filtration membrane to diminish biofilm growth.

This article provides data on the quality of water samples after different usage intervals of a newly-developed generation of terminal tap water filters.

## Methods

We decided to conduct surveillance of water samples in an intermediate care unit of the thoracic surgery department. Medical staff and the cleaning personnel on this ward are used in the general handling of tap water filters. After determination of the baseline quality of water, designated water outlets for patient care on this unit were provided with either a modified terminal water filter with a pore size of 0.2 μm (Germlyser, Aqua-Free Membrane Technology GmbH, Hamburg, Germany) or with the previous model of this water filter [19] for comparison. In contrast to the preceding model, this new water filter type is supplied with a silver layer on the filtration membrane. This silver layer is supposed to diminish growth of bacterial biofilm [20,21]. Although intended primarily for re-use after thermal disinfection [22], we only tested the water filters (old and new generation) for their performance in "first use" in this survey without consecutive reprocessing.

First, water samples were drawn from 15 water taps before installation of filters to determine the burden of pathogens in the plumbing system (baseline value). Then these 15 faucets were provided with terminal filters; 5 taps with the older product and 10 taps with the newly-modified device. Testing of filters was randomized and blinded since both generations of filters were indistinguishable in appearance. Neither the staff on the ward nor were the laboratory investigators or the physicians in the infection control department that conducted the study were aware of the type of filter. After providing the taps with filters, we examined water samples after 1, 2, 3, and 4 weeks of filter usage. Because the entire experiment was performed 4 times we repeatedly used all 15 water taps throughout the study as described above ending up in 60 samples of unfiltered water. In addition, 20 filtered water samples (old product) and 40 filtered water samples (new product) were tested after 1, 2, 3, and 4 weeks of use each (corresponding to 4 cycles with 5 and 10 samples respectively).

All water samples contained mixed (hot and cold) splash water (immediately collected when opening the water tap and starting the water flow) as we believe it is usually used for patient care in a sample volume of 500 mL. We did not perform heating of the water taps for decontamination before taking the sample as recommended in drinking water regulations, but rather adjusted our procedure to the suggestions for water surveillance in hospitals according to Pitten et al. [23].

Laboratory testing of water samples was performed on *Legionella *selective medium (GVPC agar, Oxoid, Wesel, Germany) at 36°C for 7 days as performed in previous studies and described elsewhere [19]. Total bacterial count and identification of specifically relevant pathogens such as coli-like bacteria (MacConkey agar, Oxoid), *Pseudomonas aeruginosa *(Cetrimid agar, Oxoid), or *enterococci *(Slanetz-Bartley agar, Oxoid) were also performed according to the German drinking water regulations at 22°C for 72 hours and at 36°C for 48 hours respectively [24]. Filtration technique was used to culture *P. aeruginosa*. Pour-plating technique was used to culture other bacteria for the total bacterial count. The total bacterial count was then performed quantitatively by visual examination. Chemical analysis of water samples (e.g. for heavy metal concentration of for the amount of residual disinfectants) was not performed in our study. We decided to concentrate on microbiological findings first before testing to what extent silver ions dissolve in the water.

Statistical analysis was performed applying Fisher's exact test (p < 0.05) using SPSS^® ^software. An approval of the local ethics committee for this trial was not required because the use of tap water filters is limited to intensive care units and the haematological department in our facility. Thus no other filter systems got replaced during our investigation when testing this new filter device.

## Results

### Sample size

A total of 299 water samples were analyzed during this study. At one water tap, on which a new filter was in place, we did not draw a sample after 4 weeks of use because leakage of the adapter of this particular filter was observed. Laboratory contamination of another water sample drawn from a new filter after 3 weeks of usage occurred at incubation at 22°C. All other samples were processed as described. Table 1 shows the corresponding laboratory test results and statistical analyses.

**Table 1:** Growth of *Legionella *spp. and total bacterial count (p-value compares results of water samples from old filters to results from new filters)

		number of Legionella positive samples			number of samples >100 CFU per mL in total bacterial count at 22°C			number of samples >100 CFU per mL in total bacterial count at 36°C		
unfiltered water (n = 60)		29 (48.3%)			18 (30.0%)			33 (55.0%)		

		old filter (n = 20)	new filter (n = 40)	p-value	old filter (n = 20)	new filter (n = 40)	p-value	old filter (n = 20)	new filter (n = 40)	p-value

filtered water at different usage intervals	7 days	0 (0.0%)	1 (2.5%)	1.000	3 (15.0%)	0 (0.0%)	0.033	2 (10.0%)	0 (0.0%)	0.107
	
	14 days	0 (0.0%)	0 (0.0%)	1.000	9 (45.0%)	10 (25.0%)	0.146	4 (20.0%)	8 (20.0%)	1.000
	
	21 days	0 (0.0%)	0 (0.0%)	1.000	10 (50.0%)	18 (46.2%)**	0.787	9 (45.0%)	20 (50.0%)	0.788
	
	28 days	0 (0.0%)	0 (0.0%)*	1.000	9 (45.0%)	25 (64.1%)*	0.177	14 (70.0%)	27 (69.2%)*	1.000

* Only 39 samples were tested due to leakage of the adaptor between one filter and its water tap.

** Only 39 samples were tested due to laboratory contamination during processing of one sample.

### Legionella

29 of 60 unfiltered water samples (48.3%) were *Legionella *spp. positive. *L. pneumophila *serogroup 1 was detected in 12 samples and *Legionella *of other serogroups (2 through 14) were detected in 15 samples. In 2 samples *L. pneumophila *of serogroup 1 and also of other serogroups were identified. In contrast, filtered water samples did not show growth of *Legionella spp*. except one single sample that contained 4 CFU per mL of *L. pneumophila *serogroup 1. No significant differences between different kinds of filters were observed (Table 1).

### Other relevant pathogens

*E. coli *or coli-like bacteria were not found in any sample. Growth of *P. aeruginosa *was observed in 3 unfiltered water samples (1 – 10 CFU per mL). In addition, 2 unfiltered water samples were positive for *Enterococcus *spp. All filtered water samples were negative for any of these potential relevant pathogens.

### Total bacterial count

As shown in Table 1, water samples from both kinds of filters, old and new, showed increased proportion of samples that had >100 CFU/mL over time. However, no water sample from the new generation filter system exceeded the concentration of 100 CFU per mL after 1 week of use at either incubation temperature, compared to 15% (at 22°C; p = 0.033) and 10% (at 36°C; p = 0.107) of water samples from the older filter device. After 2 weeks of use, still, there was a tendency that fewer samples were contaminated using the new filter (25%) compared with samples from the preceding model (45%) at 22°C (p = 0.146), but at incubation temperature of 36°C the level of contamination was equal (20% each; p = 1.000). No significant differences in the total bacterial count were noticed between the filter systems after a usage interval of 3 or 4 weeks at either incubation temperature.

## Discussion

For all medical departments that take care of highly immunocompromised patients, it is obligatory that effective infection control measures are implemented to prevent nosocomial Legionellosis. This may be done by attempting to eradicate pathogens from the water system or by exposition prophylaxis (avoiding contact to pathogens) as described. Because they represent a secure and convenient method to achieve *Legionella*-free water, we agree that point-of-use filtration by terminal tap water filters in high risk areas are a practical alternative of an infection control strategy to reduce the risk of nosocomial infection. In the data presented in this study, both types of filters eliminated with a single exception *Legionella *and other relevant water-borne pathogens effectively.

Unlike a recently published study testing this device [22], in our experiments the silver-layer inserted in the newly-modified filter led to a significant decrease of the total bacterial count after the first week of use only. This is most probably due to retrograde contamination during use. As noticed in earlier investigations on terminal tap water filters [18,19,25,26], retrograde contamination is a critical issue over time – even if staff on the ward is used to handling of these devices. Retrograde contamination of the filtration membrane may occur by either splash water from the water basin during use or by direct contact with contaminated hands and dirty cloths from cleaning staff. Therefore regular education of personnel (especially those who are cleaning the washing basins) and detailed instructions to patients are necessary to reduce the risk of unintended retrograde bacterial contamination. Alcohol-based surface disinfection as a routine measure and in the case of visible contamination may further reduce the risk of biofilm growth as recommended by others [22].

One limitation of our study is that we cannot rule out that retrograde contamination has occurred. As a consequence of the above-mentioned study by Daeschlein *et al*. [22], in which this type of point-of-use filter was examined, a usage interval of 4 weeks in high risk areas and 8 weeks in moderate infectious areas was recommended by the manufacturer. In their study, the water filters were automatically reprocessed in a washer/disinfector and dried at 115°C with sterile air. The total bacterial count remained remarkably low (<40 CFU per mL at 22°C as well as 36°C) over 8 weeks of use. Nevertheless, the exact clinical impact of other so-called "non-pathogenic" bacteria in the water samples in the subsequent risk of infection for highly immunocompromised patients is yet unknown. Another limitation of our study is that we only checked for the performance of the water filters in the first week of use but not after reprocessing. This practice was chosen because we primarily wanted to assess the baseline quality of a new product that was still within the development process at that time.

Oyanedel-Craver *et al*. used colloidal-silver-impregnated ceramic filters for point-of-use water treatment and determined silver concentrations in the effluent water; the values were initially >0.1 mg/L but dropped below this value after continuous operation [27]. Others confirmed that this concentration is effective in killing pathogens [28] and the WHO considers this concentration being safe for human health [29]. Unfortunately, as another limitation of the present study, neither we nor Daeschlein *et al*. [22] measured the concentration of silver ions in the water samples in the Germlyser filter device.

## Conclusion

We are well aware of the presence of *Legionella spp*. in our hospital water supply system and point-of-use filtration has been the method of choice for high risk patient care in our facility for years. Since the beginning of active surveillance in 2000, to our knowledge, no cases of nosocomial Legionellosis have occurred in our hospital. That is, why we and others [30] propose using tap water filter primary for the prevention of nosocomial Legionellosis for high risk patient groups. The use of anti-bacterial components in the manufacturing process of water filters might be helpful for the prevention of biofilm growth and the extension of the usage interval of such devices.

## List of abbreviations

CFU: colony forming unit; HICPAC: Healthcare Infection Control Practices Advisory Committee; CDC:Centers for Disease Control and Prevention; IDSA: Infectious Diseases Society of America; ASBMT: American Society of Blood and Marrow Transplantation;

## Competing interests

Funding of € 10.000 was received from Aqua-Free Membrane Technology GmbH, Hamburg, Germany, to conduct this study.

## Authors' contributions

Water sampling and microbiological evaluation was carried out by RPV, JB, and PG. DS performed statistical analysis of the results. The manuscript was written by RPV and PG. All authors read and approved the final manuscript.

## Pre-publication history

The pre-publication history for this paper can be accessed here:


